# Peripherally-administered amphetamine induces plasticity in medial prefrontal cortex and nucleus accumbens in rats with amygdala lesions: implications for neural models of memory modulation

**DOI:** 10.3389/fnbeh.2023.1187976

**Published:** 2023-06-08

**Authors:** Robert J. McDonald, Nancy S. Hong, Carlie Germaine, Bryan Kolb

**Affiliations:** Canadian Centre for Behavioural Neuroscience, Department of Neuroscience, University of Lethbridge, Lethbridge, AB, Canada

**Keywords:** amygdala, amphetamine, plasticity, memory modulation, drugs of abuse

## Abstract

The amygdala has been implicated in a variety of functions linked to emotions. One popular view is that the amygdala modulates consolidation in other brain systems thought to be mainly involved in learning and memory processes. This series of experiments represents a further exploration into the role of the amygdala in memory modulation and consolidation. One interesting line of research has shown that drugs of abuse, like amphetamine, produce dendritic changes in select brain regions and these changes are thought to be equivalent to a usurping of normal plasticity processes. We were interested in the possibility that this modulation of plasticity processes would be dependent on interactions with the amygdala. According to the modulation view of amygdala function, amphetamine would activate modulation mechanisms in the amygdala that would alter plasticity processes in other brain regions. If the amygdala was rendered dysfunctional, these effects should not occur. Accordingly, this series of experiments evaluated the effects of extensive neurotoxic amygdala damage on amphetamine-induced dendritic changes in the nucleus accumbens and prefrontal cortex. The results showed that rats with large lesions of the amygdala showed the normal pattern of dendritic changes in these brain regions. This pattern of results suggests that the action of not all memory modulators, activated during emotional events, require the amygdala to impact memory.

## 1. Introduction

The amygdala is a neural system important for emotions (Kluver and Bucy, 1939), learning and memory (Weiskrantz, 1956; Goddard, 1964; Bagshaw and Benzies, 1968; Davis et al., 1982; Cador et al., 1989; Everitt et al., 1989; Hiroi and White, 1991), attentional processes (Gallagher et al., 1990), and modulation of plasticity processes underlying memory during emotional events (McGaugh et al., 1996). Human studies have provided similar evidence supporting the view that the amygdaloid complex is the locus of affective memory formation and storage (Breiter et al., 1996; Cahill et al., 1996; Irwin et al., 1996; Reiman et al., 1997; Whalen et al., 1998; Cheng et al., 2003).

Despite the fact that there is little debate about the role of the amygdala in emotionally-related brain functions (see White and McDonald, 1993; McGaugh, 2002), there is muted debate about the specific role of the amygdala in these processes (Cahill and McGaugh, 1998; Fanselow and LeDoux, 1999; Wilensky et al., 2000; Antoniadis and McDonald, 2001; Wallace and Rosen, 2001; Holahan and White, 2002; McDonald et al., 2007a). One point of contention is whether the amygdala is a central module of a complex neural circuit supporting emotional learning and memory functions or whether the amygdala simply modulates other learning and memory systems during biologically significant events. The former is sometimes referred to as the *storage view* while the latter is the *modulation view* of amygdala function.

In support of the storage view, the amygdala has been implicated in different but related functions including stimulus-reward learning. This view suggests that various nuclei of the amygdala are essential for the acquisition and storage of stimulus-reward associations (Schwartzbaum, 1960, 1964, 1965; Schwartzbaum and Poulos, 1965; Spiegler and Mishkin, 1981; Gaffan and Harrison, 1987; Gaffan et al., 1989; Everitt et al., 1991; Kentridge et al., 1991; McDonald and White, 1993; McDonald et al., 2010). The amygdala has also been implicated in aversive classical conditioning (Bagshaw and Benzies, 1968; Kapp et al., 1979; Davis, 1986; Ledoux et al., 1990; Kim et al., 1993; Antoniadis and McDonald, 2000; Maren, 2003). This information is then used to guide decision making and select behaviors via direct interactions with prefrontal cortex and dorsal and ventral striatum (Mulder et al., 1998; Baxter et al., 2000; Gruber and McDonald, 2012).

One logical reason why the amygdala plays such a fundamental role in emotional learning and memory processes is the unique and extensive reciprocal connectivity of this structure to both cortical and subcortical targets. Briefly, the amygdala receives extensive sensory input from the thalamus and/or cortex from all of the major sensory modalities (for review see White and McDonald, 2002). The feature of amygdala connectivity suggests that it has online access to information about the external environment. The amygdala also has extensive reciprocal connections with portions of the hypothalamus, brainstem, and ventral striatum. These brain areas control functions like heart rate, respiration, hormone release, and neurotransmitter release that occur during negative and positive experiences. This feature of amygdala anatomy provides the system with information about negative and positive events (Braesicke et al., 2005). In combination, sensory and affective information converge in the amygdala and plasticity mechanisms contained within this structure forms associative memories of this information for later use.

According to the modulation view, the main function of the amygdala is to modulate plasticity processes, initiated during biologically significant events, thus enhancing these experiential changes in the brain (McGaugh et al., 1993; Cahill and McGaugh, 1998; Packard and Cahill, 2001). The idea is that arousal associated with a biologically significant experience activates the amygdala via stress hormone release like norepinephrine and corticosterone as well as activate other neurotransmitter systems like dopamine (Gasbarri et al., 1997). The amygdala then modulates plasticity processes in various areas of the brain encoding a particular experience. Specifically, evidence has been put forth suggesting that noradrenergic activation of the amygdala may serve to modulate memory storage and plasticity in the hippocampus (Ikegaya et al., 1997; Ferry et al., 1999; Frey et al., 2001) including long-term potentiation in perforant path/dentate gyrus (Akirav and Richter-Levin, 2002). This idea is also supported by studies showing time-dependent involvement of the amygdala on an inhibitory avoidance task (Liang et al., 1982), and enhanced performance with post-training intra-amygdala infusions of d-amphetamine on the spatial version of the water maze task (Packard et al., 1994).

We have previously challenged the modulation view of amygdala function (McDonald et al., 2007a) suggesting, based on our empirical evidence, that although the amygdala does modulate memory processes in other memory networks, these effects might be secondary to its emotional learning and memory functions. The experiments showed that the functions of the amygdala were not necessary for normal hippocampal learning and memory processes including tasks that evaluated the effects of task difficulty and long-decay rates. Furthermore, rats with large amygdala lesions showed normal post-training memory improvement effects on a hippocampal-based navigational task. Despite the lack of effects of amygdala damage on modulation of hippocampal learning and memory processes, the rats without a functional amygdala were impaired on the acquisition of a fear-based context conditioning task. An important implication of this pattern of results, which is the focus of the current experiments, is that it appears that there are multiple memory modulation pathways in the mammalian brain, some of which are completely independent of the amygdala.

The goal of the present experiments was to provide further empirical tests of the idea that the amygdala modulates plasticity processes in distal brain regions. One biologically significant event of interest to our research group is the experience of taking drugs of abuse. Repeated administration of many drugs of abuse result in neural changes in brain regions, like prefrontal cortex and nucleus accumbens, that might be mediated via dopamine receptors (Robinson and Kolb, 1997). One mechanism for the long-term behavioral sensitization observed following repeated drug administration is structural modifications in neural circuits implicated in drug abuse, including the nucleus accumbens and prefrontal cortex (Robinson and Kolb, 2004). It has been shown that repeated amphetamine treatment produces changes in neuronal morphology including long-lasting increases in dendritic length, dendritic density, and number of spines (Robinson and Kolb, 1997). Importantly, amphetamine has also been shown to be a powerful modulatory of memory and has been used for 30 decades in conjunction with the post-training memory improvement paradigm for this purpose and presumably dependent on the amygdala (Packard et al., 1992).

The present study will determine if the dendritic changes found in the nucleus accumbens and prefrontal cortex following repeated administration of amphetamine are dependent on modulation functions of the amygdala. According to the modulation view of amygdala function, the dendritic changes should be occluded in rats with amygdala damage.

## 2. Materials and methods

### 2.1. Subjects

Twelve male Long-Evans rats (300–350 g) from the University of Lethbridge breeding program were used for the present study. The rats were paired housed and maintained on a 12:12 light/dark cycle and had food and water available to them *ad libitum*. All rats were handled by the experimenter for 5 min per day for 4 days prior to surgery.

### 2.2. Surgery

All surgical procedures were performed in accordance with the rules and guidelines set by the Canadian Council on Animal Care and the University of Lethbridge animal welfare committee. Six animals underwent bilateral basolateral amygdala lesions, and five received sham control surgery. Surgery was conducted while rats were anesthetized with Isofluorane anesthesia (4% with 2 L/min of oxygen for induction and 2% after surgical plane was established) in a standard stereotaxic apparatus. Due to the fact that lesions to the amygdala produce seizures, the rats were also given 0.2cc of Sodium Pentobarbital and 0.1cc of Diazepam (both administered *i.p.*) prior to surgery. Once under anesthesia, the hair from on top of the rat’s head was shaved and the skin cleaned with hibitane and alcohol. An incision was made in the scalp and periosteum along the midline. The fascia (periosteum) was cut laterally across the top of the skull and pushed to the edges of the surgical site with a sterile gauze swab. The skin was retracted with 2 mosquito hemostats to expose the skull surface and trephining holes were drilled into the skull using a 2mm drill bit. The coordinates (in millimeters relative to bregma) for basolateral amygdala lesions were: AP −2.3, −3.3; ML ± 4.8, 4.6; DV −9.4, −9.4. Lesions were produced with NMDA (5 mg/ml) and were infused through 30-ga cannulae attached to a Harvard mini-pump. The infusion rate was 0.2 μl per min for 4 min. After each infusion, the cannulae were left in place for a 2-min diffusion period. Upon completion the skin was sutured using 4.0 sterile sutures, and Buprenorphine (0.05 mg/kg) was given (subcutaneously) as a post-operative analgesic. The sham group underwent the same surgical procedure except no infusions were performed. We did not include a sham infusion group as we have done this control infusion procedure repeatedly and never found a functional effect. Thus, we are convinced it is not necessary. This decision was also influenced by recommended reductions in the amounts of subjects used for research whenever possible coming from federal animal care agencies. Animals were given 1 week to recover from surgery prior to experimentation.

### 2.3. Amphetamine exposure

Half of the animals from the amygdala lesion group (*n* = 3 of 6) and half from the sham group (*n* = 3 of 6) were treated with D-amphetamine sulfate (Sigma Aldrich, St. Louis, MO, USA), a highly addictive psychomotor stimulant, previously shown to induce significant dendritic changes in various parts of the brain (Robinson and Kolb, 1997). Each rat in the AMPH group received repeated exposure to D-amphetamine sulfate (1 mg/kg) dissolved in sterile 0.9% saline. The rest of the lesion animals (*n* = 3 of 6) and sham controls (*n* = 3 of 6) were given sterile 0.9% saline injections.

Both AMPH and saline groups were administered *i.p.* at a volume of 1 ml/kg. Rats were immediately placed back in the activity boxes post-injections and the activity was recorded for 90 min. The drug was administered once a day for 10 consecutive days at approximately the same time every day. Locomotor activity recorded on a computer with VersaMax program was converted to spreadsheets using VersaDat software (AccuScan Instruments, Inc). The rats were returned back to their home cages each day after the end of AMPH testing session. Locomotor activity was assessed every 2nd day of drug administration (Day 2, 4, 6, 8, 10). Unfortunately, it was not until the activity data was pulled from the computer that we realized there was a technical issue with some of the apparatus resulting in the data being uninterpretable in the sham control groups on Day 4, 6, 8, and 10. Also, one of the sham controls given saline injections became ill 2 weeks following completion of injections and was humanely euthanized. The activity data recorded for this animal after Day 2 injection is not included in the results.

### 2.4. Histology

Thirty days after the last treatment with amphetamine or saline, the rats were deeply anesthetized with sodium pentobarbital and then perfused intracardially with 0.9% saline. The rats were perfused after 30 days to be consistent with most of our earlier studies. Robinson and Kolb (2004) have assessed the temporal aspects of these effects in many publications and the changes occur within 24–48 h and can last for over 4 months and the amount of change is not different either. This suggests that the dendritic changes are not simply due to the 30-day delay and potential withdrawal effects.

The brains were extracted and prepared for Golgi–Cox staining. Traditional Golgi–Cox methods provide capricious staining of spines, but the modified method used here allows consistent visualization of spines (Kolb and McClimans, 1987; Kolb et al., 1994). Briefly, the brains were first placed in Golgi–Cox solution for 14 days followed by 3 days in 30% sucrose. The brains were then cut using a vibratome into 200 μm coronal sections, with every section in the cerebral hemispheres saved and stained. The neurons were drawn from 2 to 3 sections through the regions in question and 10 cells were selected from each animal. The entire cell was drawn. Cells that were obviously incomplete (i.e., part of the cell was truncated) because of the angle of section were not drawn. To be included in the analysis, the dendritic tree of a cell had to be well impregnated and not obscured with stain precipitations, blood vessels, or astrocytes, and the dendritic fields had to appear primarily intact and visible in the plane of section (Kolb and McClimans, 1987). The relevant brain regions were identified at low power (100×), and five layer III pyramidal cells from each hemisphere were drawn using a camera lucida (at 250×) in cortical area Cg 3 (prefrontal) as defined by Zilles (1985). Medium spiny neurons in the nucleus accumbens were identified and drawn in the same manner. A Sholl analysis (Sholl, 1981) of ring intersections was used to estimate dendritic length. A length of dendrite (>10 μm) was traced (1000×), and the exact length of the dendritic segment was calculated. Cell selection and drawing were performed by a person blind to treatment conditions. Each hemisphere represented a “subject” so that two subjects were obtained per rat (Robinson and Kolb, 1997). The results from experiments that use hemisphere as the subject in these kinds of experiments has been reliably replicated repeatedly over the decades (Robinson and Kolb, 2004).

Statistical analyses were performed by averaging across cells per hemisphere, and group differences were assessed using ANOVA and *post-hoc* tests when appropriate.

## 3. Results

### 3.1. Histology

#### 3.1.1. Amygdala lesions

**FIGURE 1:**
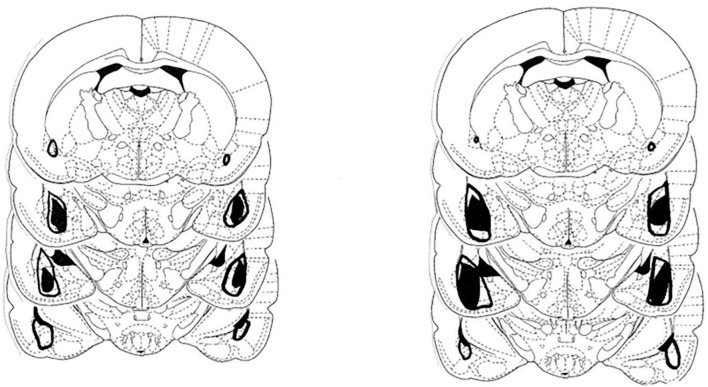
Depiction of the largest (open area) and smallest extent (darkened area) of the neurotoxic lesions directed at the amygdala in the group that received saline injections **(left)** and the treatment group that received amphetamine **(right)**.

The extent of the amygdala damage in the two lesion groups is shown in Figure 1. As can be seen there was extensive damage to both the anterior and posterior portions of the amygdala. The damage included large portions of the basolateral, lateral, and central amygdala nuclei. The extent of the amygdala damage in this study is consistent with our previous behavioral work using this methodology.

#### 3.1.2. Amphetamine induced activity

**FIGURE 2:**
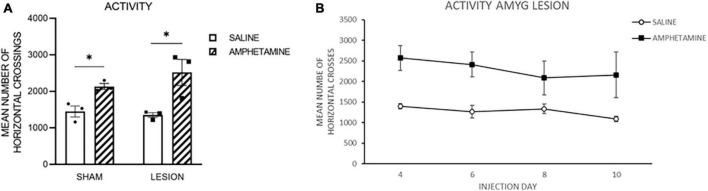
**(A)** The amount of activity following injections of amphetamine (amph) or saline in the sham and amygdala lesion (lesion) groups on Day 2. As is evident in this figure, both groups that received amph injections exhibited increased activity as compared to the groups receiving saline. **(B)** The activity for the lesion group following injections of saline or amph on Day 4, 6, 8, and 10. As can be seen, the lesion group injected with amph had more activity than those injected with saline, and this difference was evident throughout the days of drug injection. The *symbol indicates that the groups were statistically different.

As indicated previously, there was a technical issue with some of the boxes in the sham control group and therefore the results are presented for Day 2 of drug injection for all of the groups, and then only for the amygdala lesion (lesion) groups. Figure 2A represents the amount of activity following injections of AMPH or saline in the sham and lesion groups on Day 2. As is evident in this figure, both groups that received AMPH injections exhibited increased activity as compared to the groups receiving saline. This was confirmed by a 2-way ANOVA with fixed effects indicating a significant effect of Drug [*F*_(1,7)_ = 13.90, *p* < 0.008], but no effect of Group [*F*_(1,7)_ = 0.40, *p* = 0.55] nor interaction [*F*_(1,7)_ = 0.37, *p* = 0.56]. Figure 2B shows the activity for the lesion group following injections of saline or AMPH on Day 4, 6, 8, and 10. As can be seen, the lesion group injected with AMPH had more activity than those injected with saline, and this difference was evident throughout the days of drug injection. A Two-way ANOVA with repeated measures on Day verified this impression indicating a significant effect of Drug [*F*_(1,4)_ = 8.48, *p* = 0.04], but no Day *F*_(3,12)_ = 1.39, *p* = 0.29] nor interaction effect [*F*_(3,12)_ = 0.54, *p* = 0.66].

#### 3.1.3. Dendritic branching

**FIGURE 3:**
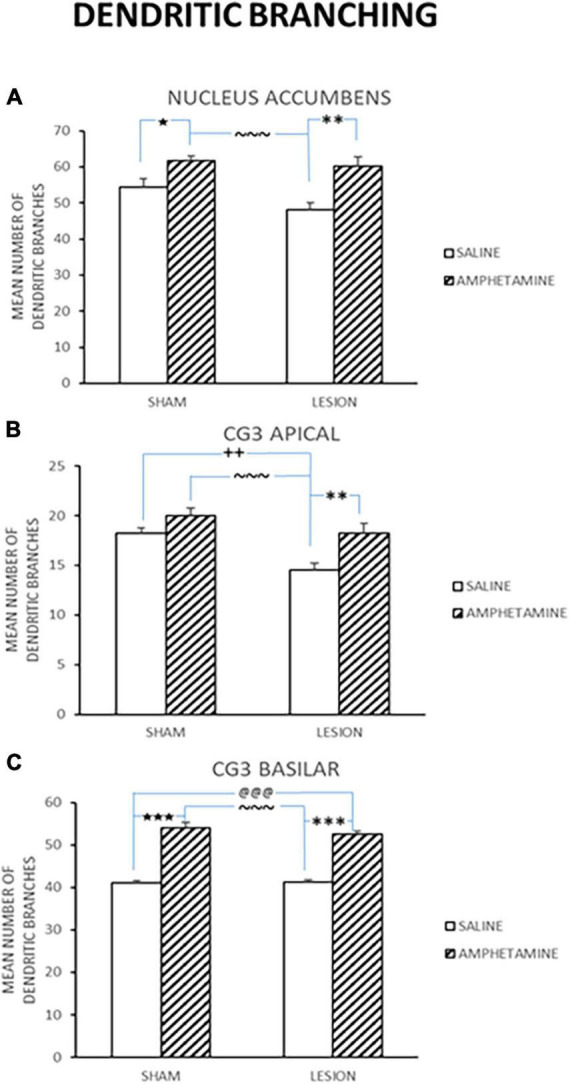
Mean number of dendritic branching (±SE) in sham and lesion rats injected with saline or amphetamine (amph) in panel **(A)** nucleus accumbens; **(B)** Cg3 apical; and **(C)** Cg3 basilar is shown. Both the sham and lesion groups treated with amph had greater increases in dendritic branching in all these regions. Significant differences are represented by ^★^between the sham/saline and sham/amph groups; ^@^between the sham/saline and lesion/amph groups; *between the lesion/saline and lesion/amph groups; ^∼^between the sham/amph and lesion/saline groups; ^+^between the sham/saline and lesion/saline groups. A single symbol represents 0.05 < *p* > 0.01; two of the same symbol represents 0.01 < *p* > 0.001; three of the same symbol represents *p* < 0.001.

**FIGURE 4:**
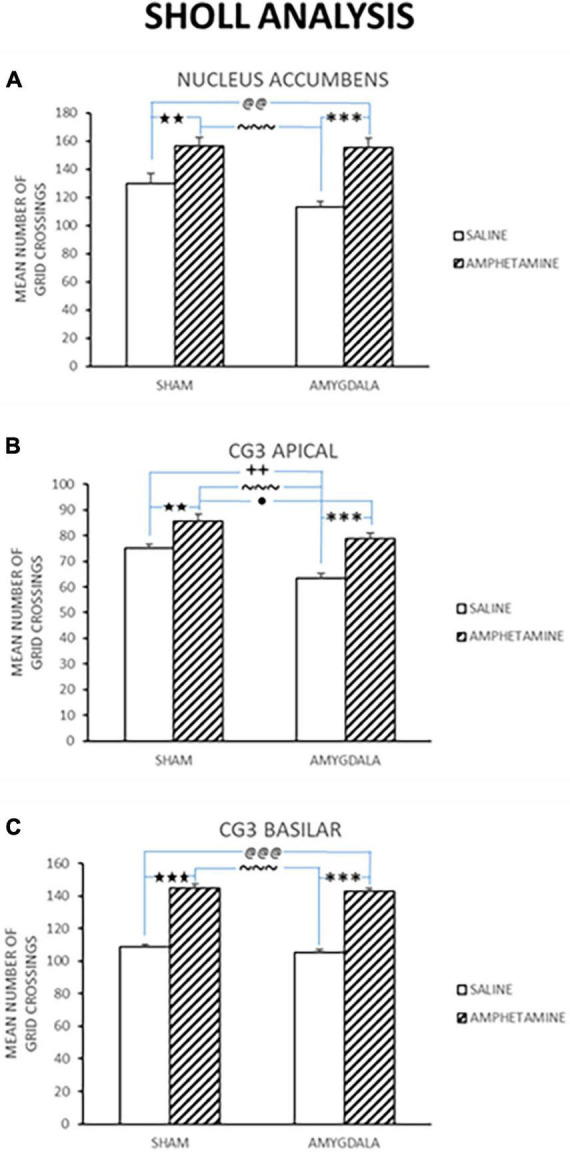
Dendritic length was estimated using a Sholl analysis, and thus the numbers indicate ring intersections. Mean dendritic length (±SE) in sham and lesion rats injected with saline or amphetamine (amph) in panel **(A)** nucleus accumbens; **(B)** Cg3 apical; and **(C)** Cg3 basilar is shown. In all of these regions, the groups treated with amph show increased dendritic length compared to groups given saline. Significant differences are represented by ^★^between the sham/saline and sham/amph groups; ^@^between the sham/saline and lesion/amph groups; *between the lesion/saline and lesion/amph groups; ^∼^between the sham/amph and lesion/saline groups; ^+^between the sham/saline and lesion/saline groups; ^●^between the sham/lesion and lesion/amph groups. A single symbol represents 0.05 < *p* > 0.01; two of the same symbol represents 0.01 < *p* > 0.001; three of the same symbol represents *p* < 0.001.

Thirty days following the last day of treatment administration, the rats were sacrificed and brains assessed for changes in dendritic branching and length. Mean counts of various brain areas, including the nucleus accumbens (Nacc), cingulate cortex (CG3) apical, and CG3 basilar, in the same rats were examined for dendritic branching (Figure 3) and dendritic length (Sholl) (Figure 4) changes. As can be seen, there were significant differences between the treatment groups on some of these measures. Figure 3A shows the mean number of dendritic branching of medium spiny neurons in the nucleus accumbens of rats with NMDA-induced amygdala lesions (AMYG) or sham control rats that were injected with either saline or amphetamine (AMPH). As can be seen, the amygdala and sham control groups that were given amphetamine showed an increase in dendritic branching. A quantitative analysis verified this impression as groups treated with AMPH had greater increases [*F*_(3,18)_ = 9.464, *p* < 0.001]. Fisher’s PLSD indicated significant differences when comparing the sham/saline and sham/AMPH groups (*p* = 0.032); the AMYG/saline and AMYG/AMPH groups (*p* = 0.0005); and the sham/AMPH and AMYG/saline groups (*p* < 0.0001). No differences were found between the sham/saline and AMYG/saline groups; the sham/saline and AMYG/AMPH groups; or the sham/AMPH and AMYG/AMPH groups (*p’*s > 0.05). These results suggest that groups given amphetamine have increased dendritic branching in the nucleus accumbens and that this occurs in sham control as well as amygdala lesion rats. Furthermore, sham control rats are not different from amygdala lesion rats in the number of dendritic branches in the nucleus accumbens. Figure 3B displays dendritic branching of apical prefrontal cortex neurons in layer III (Cg3) from sham control and amygdala lesioned rats given saline or AMPH. As can be seen in this graph, the groups given AMPH had greater dendritic branching than the groups given saline. This observation was confirmed by a quantitative analysis indicating a significant treatment effect [*F*_(3,18)_ = 8.918, *p* < 0.001]. Comparisons using Fisher’s PLSD showed significant differences between the sham/AMPH and AMYG/saline groups (*p* < 0.0001); the AMYG/saline and the AMYG/AMPH groups (*p* = 0.0032); and sham/saline and AMYG/saline groups (*p* = 0.0068). No differences were found between the sham/saline and the sham/AMPH groups, the sham/saline and the AMYG/AMPH groups, or the sham/AMPH and AMYG/AMPH groups (*p*’s > 0.05). These results suggest that AMPH increases dendritic branching in apical Cg3 neurons, however this effect was more prominent in the amygdala lesion group than sham controls. Dendritic branching of basilar Cg3 neurons is displayed in Figure 3C. As illustrated, the groups treated with AMPH show increased dendritic branching in this region compared to those treated with saline. An analysis reported a significant treatment effect [*F*_(3,18)_ = 72.624, *p* < 0.0001]. Significant differences using Fisher’s PLSD were found between the sham/saline and sham/AMPH groups (*p* < 0.0001); the sham/saline and the AMYG/AMPH groups (*p* < 0.0001); NMDA/saline and sham/AMPH groups (*p* < 0.0001); and AMYG/saline and AMYG/AMPH groups (*p* < 0.0001). No differences were present between the sham/saline and AMYG/saline groups or the sham/AMPH and AMYG/AMPH groups (*p*’s > 0.05) suggesting that dendritic branching in Cg3 neurons was comparable in rats with amygdala lesions and controls. Further, treatment with amphetamine significantly increased spine density in basilar Cg3 neurons regardless of whether rats had amygdala lesions or not.

#### 3.1.4. Dendritic length

**FIGURE 5:**
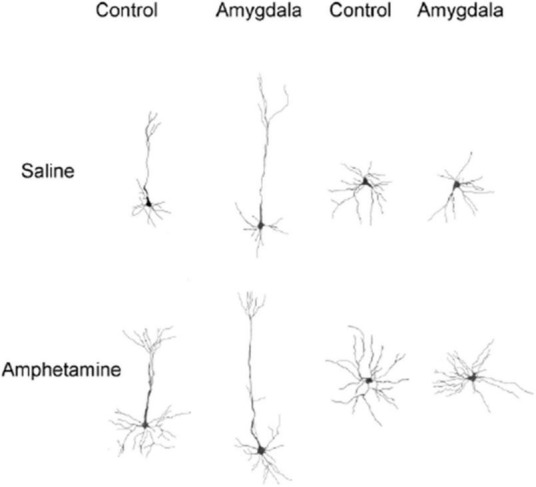
Camera lucida drawings of representative neurons from control and amygdala rats treated with saline or amphetamine. Left side drawings are of layer III pyramidal neurons in Cg 3. Right side drawings are of medium spiny neurons in nucleus accumbens. Cells from rats receiving amphetamine have increased branching and dendritic length compared to cells from control rats.

Dendritic length was estimated using a Sholl analysis and thus the numbers indicate ring intersections. Dendritic length data collected from the nucleus accumbens in control and amygdala lesion rats treated with saline or AMPH is depicted in Figure 4A. This figure shows that there was an increase in dendritic length in the sham control and amygdala lesioned rats that were injected with amphetamine. A quantitative analysis reported a significant treatment effect [*F*_(3,18)_ = 14.222, *p* < 0.0001]. Fisher’s PLSD confirmed significant differences between the sham/saline and sham/AMPH groups (*p* < 0.007); sham/saline and AMYG/AMPH groups (*p* < 0.009); sham/AMPH and AMYG/saline groups (*p* < 0.0001); and AMYG/saline and AMYG/AMPH groups (*p* < 0.0001). No differences were found between the sham/saline and the AMYG/saline groups; nor the sham/AMPH and the AMYG/AMPH groups (*p*’s > 0.05) suggesting that dendritic length is not significantly different in amygdala lesion rats compared to sham control rats. Rather, increases in dendritic length in the nucleus accumbens were greater with the administration of amphetamine than saline in both the sham control and amygdala lesion groups. Dendritic length in apical Cg3 neurons of sham controls and rats with amygdala lesions, given saline or amphetamine, were also examined and are shown in Figure 4B. As observed in this figure, the groups given amphetamine have increased dendritic length than those given saline. A quantitative analysis yielded a significant treatment effect [*F*_(3,18)_ = 17.883, *p* < 0.0001]. Significant differences were obtained using Fisher’s PLSD when comparing the sham/saline and sham/AMPH groups (*p* < 0.01), the sham/saline and AMYG/saline groups (*p* < 0.002); the sham/AMPH and the AMYG/saline groups (*p* < 0.0001); the sham/AMPH and AMYG/AMPH groups (*p* = 0.04); and the AMYG/saline and AMYG/AMPH groups (*p* < 0.0001). No differences were found between the sham/saline and AMYG/AMPH groups (*p* > 0.05). These results suggest that increases in dendritic length in apical Cg3 neurons were more prominent in the sham control group than the rats with amygdala lesions, as well as with the administration of amphetamine in these two groups. Figure 4C displays the dendritic length in basilar Cg3 neurons of sham control and amygdala lesion rats given saline or amphetamine. As can be seen, the groups treated with amphetamine show increased dendritic length. This was verified with a quantitative analysis indicating a significant treatment effect [*F*_(3,18)_ = 93.84, *p* < 0.0001]. Fisher’s PLSD showed significant differences between the sham/saline and sham/AMPH (*p* < 0.0001); the sham/saline and AMYG/AMPH groups (*p* < 0.0001); the AMYG/saline and sham/AMPH groups (*p* < 0.0001); and the AMYG/saline and AMYG/AMPH groups (*p* < 0.0001). No differences were obtained between the sham/saline and AMYG/saline groups nor the sham/AMPH and AMYG/AMPH groups (*p*’s > 0.05). Figure 5 shows drawings of neurons (pyramidal and medium spiny neurons) randomly selected from the four experimental groups which are consistent with the dendritic changes reported in this paper. Taken together, these results suggest that dendritic length is increased with the administration of amphetamine in both the control and amygdala lesion groups. The absence of locomotor activity in the saline injected subjects due to a technical malfunction, although not ideal from a symmetry perspective, is not fatal as this data set is not required to conclude that the amphetamine subjects received the injections as the brain analysis clearly shows dendritic changes previously shown to occur following repeated exposure to amphetamine.

## 4. Discussion

These experiments were designed to determine if the amygdala contributed to the amphetamine-induced dendritic plasticity changes found in the nucleus accumbens and medial prefrontal cortex. The amphetamine manipulation is relevant as one result of these injections is a significant amount of dopamine release in the mesolimbic dopamine system, which also occurs during both positive and negative emotional experiences (Packard and Cahill, 2001; McDonald et al., 2007a; Papalini et al., 2020).

If amphetamine exposure does not result in the plastic changes in the nucleus accumbens and medial prefrontal cortex in rats with large amygdala lesions this would be consistent with the idea that the amygdala is a crucial neural nexus of all forms of memory modulation (McGaugh, 2000). However, the results showed that rats with large neurotoxic lesions of the amygdala exhibit normal patterns of dendritic changes following amphetamine exposure (Robinson and Kolb, 1997). Specifically, increases in dendritic branching and dendritic length were found in the nucleus accumbens in controls and rats with large amygdala lesions. Similar increases in dendritic branching and dendritic length were found in prefrontal cortex (CG3) neurons on both apical and basilar dendrites. This pattern of results suggests that the amygdala is not necessary for modulating plasticity processes elicited by dopamine release, following peripheral amphetamine injections, like it seems to be for stress hormones like noradrenaline and glucocorticoids (LaLumiere et al., 2017). As noted before, the mesolimbic dopamine system is activated during positive and negative emotional events alongside the stress hormone response (Ploski and McIntyre, 2015; Baik, 2020) suggesting that our amphetamine manipulation is mimicking a part of the stress hormone response found when aversive stimuli or stress hormones are manipulated in memory modulation experiments.

Another effect was found in the present experiments that is intriguing. Damage to the amygdala, on its own, resulted in some reductions in the dendritic measures assessed in the prefrontal cortex and nucleus accumbens. We are unaware of an effect like this reported in the literature. This result suggests that the amygdala does have impacts on plasticity in these regions in adulthood. One possibility is that interactions with cage-mates and animal care interventions (cage changes, etc.) are normally encoded by the amygdala network allowing the animal to learn that these events are not dangerous or unpredictable. Without an amygdala these processes would not occur and could result in an amplified stress hormone response to these events potentially altering dendritic morphology in specific brain areas. Another possibility is that modulatory effects of the amygdala are missing during these same kinds of experiences resulting in dendritic changes. It really could be interpreted as consistent with either view of amygdala function at this point. Regardless of this effect, the amphetamine manipulation continued to have similar impacts on these brain regions whether the amygdala was intact or not.

### 4.1. Amygdala does not appear to modulate plasticity processes elicited by dopamine release

The pattern of results reported in the present paper is inconsistent with the view that the amygdala modulates all plasticity processes in other neural systems that occur during emotional events. The current results are unique because dendritic changes in different brain regions were assessed following an experience in the absence of amygdala function, whereas in our previous work we evaluated the functional impacts of amygdala dysfunction on learned behaviors mediated by different learning and memory systems (McDonald et al., 2007a). Briefly, in that study we showed that rats with large neurotoxic lesions of the amygdala showed no impairment on acquisition of versions of spatial memory tasks that differed in difficulty nor did they exhibit any alterations in memory decay rates on these hippocampal-based tasks (Whishaw, 1985; Sutherland et al., 2000). Even more surprisingly, subjects with amygdala damage showed a normal post-training memory enhancement effect on a task sensitive to hippocampal and not amygdala damage (Morris et al., 1982; Sutherland et al., 1982; Sutherland and McDonald, 1990). The results of the present experiments are consistent with this behavioral work.

### 4.2. Multiple pathways for memory modulation

The view that memory modulation processes in the mammalian brain are dependent on the amygdala (Cahill and McGaugh, 1996, 1998; Packard and Teather, 1998; McGaugh, 2002, 2005; McGaugh et al., 2002; Huff and Rudy, 2004; Holahan et al., 2005) appears to be a simplified position. Our view is that there are multiple types of memory modulation processes, one of which is dependent on interactions with the amygdala and a host of others that are not. Specifically, we propose that there are at least 6 different types of modulation that can occur following an emotionally arousing event.

Three of these potential modulation mechanisms could contribute to the dendritic plasticity changes observed following chronic exposure to amphetamine in the current experiments. First, ascending neurotransmitter systems, like dopamine, can directly influence sensory, motor, and motivational systems. Release of these neurotransmitters, from these direct projections, can interact with other neurotransmitter systems like glutamate to modulate memory formation, consolidation and related plasticity processes like dendritic changes (Kilgard and Merzenich, 1998; Bao et al., 2001; Mercado et al., 2001). A second type of memory modulation is similar to the first, except that the ascending neurotransmitter systems project directly to various neural systems, like the prefrontal cortex, and directly modulate plasticity processes underlying memory. Finally, hormonal release can also influence learning and memory systems directly by activating hormone specific receptors that are found in high concentrations in these structures. For example, glucocorticoids are released during emotional arousal and is a hormone receptor that is found throughout the brain that has been shown to modulate plasticity processes supporting memory (Roozendaal and McGaugh, 1996).

Although of general interest, other documented modulation mechanisms would probably not contribute to the dendritic plasticity changes observed following chronic exposure to amphetamine in the current experiments. One of these mechanisms is via firing patterns unique to a specific learning and memory system during certain brain states. For example, the hippocampus elicits firing patterns during certain stages of sleep that resemble firing patterns during recent learning bouts (Wilson and McNaughton, 1994). These processes are thought to mediate memory consolidation in the hippocampus. A second mechanism by which memory modulation could occur is via intrinsic neurobiological processes intrinsic to a specific learning and memory system. For example, work suggests that neurogenesis in the hippocampus plays a role in the long-term consolidation of spatial memories (Snyder et al., 2005). One possibility is that these new cells, during encoding, provide trophic factors that influence memory storage and the long-term integrity of memories intrinsic to the hippocampus. Finally, another mechanism by which plasticity and associated memories are modulated is via interactions between the amygdala and different learning and memory systems. The exact nature of these processes is still open for debate including whether these modulation effects are unconditioned or a secondary consequence of conditioning processes in the structure (Holahan and White, 2002, 2004).

**FIGURE 6:**
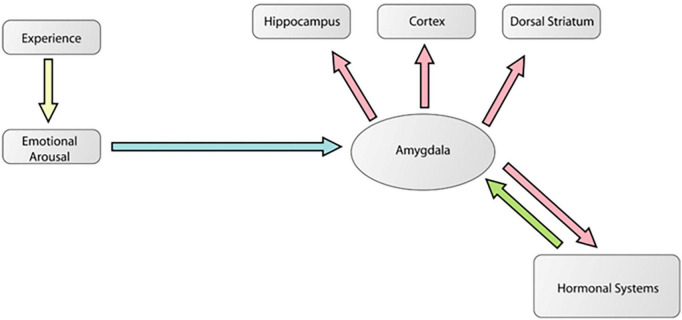
A modified diagram from a theoretical paper (Cahill and McGaugh, 1998) showing how the amygdala is an essential part of a mechanism by which emotionally arousing events modulate memory in areas like the hippocampus. In this model, arousal associated with a biologically significant experience activates the amygdala via stress hormone release like norepinephrine and corticosterone. These signals reach and activate the amygdala which then modulates plasticity processes in various areas of the brain encoding that particular experience. This is the traditional modulation view of amygdala function. This figure was reproduced from McDonald et al. (2007a).

**FIGURE 7:**
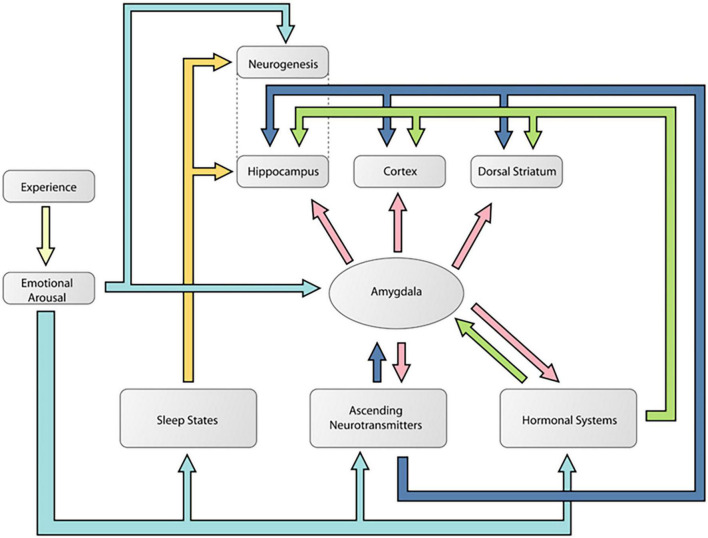
A diagram showing likely multiple pathways of memory consolidation suggested by the results of the present experiment. Note this model includes the pathways proposed by Cahill and McGaugh (1998). According to this model, an emotional experience activates multiple neural responses that can modulate learning and memory processes in different networks. These responses included noradrenaline and corticosterone, ascending neurotransmitter systems like dopamine, sleep processes, and neurogenesis. The color coding is used to delineate the different potential routes of memory modulation during an emotional event. The light blue arrows indicate how an emotional event can activate the different mechanisms of memory modulation. The dark blue arrows show how an emotional event can activate ascending neurotransmitter systems that can potentially modulate memory independent of the amygdala. The yellow arrows indicate pathways of memory modulation that might be specific to the hippocampus and do not require amygdala involvement. Finally, the pink arrows indicate potential amygdala influences on memory. This figure was reproduced from McDonald et al. (2007b).

Figure 6 is a diagram [adapted from Cahill and McGaugh (1998)] showing how the amygdala is an essential part of a mechanism by which emotionally arousing events modulate memory in areas like the hippocampus. This is the traditional modulation view of amygdala function. Figure 7 is a diagram [adapted from McDonald et al. (2007b)] showing multiple pathways of memory consolidation proposed in the present report.

One parsimonious explanation for the current results and previous research suggesting a role of the amygdala in memory modulation is the possibility that the basolateral amygdala is specifically involved in norepinephrine-mediated memory modulation as this hormone is released into the blood stream and reaches regions of the brain important for learning and memory. This appears to be accomplished via a circuit that includes the basolateral amygdala. Various experiments have shown that norepinephrine released in the blood stream during an emotionally arousing event access the brain via norepinephrine receptors found on the vagus nerve. Once the vagus is activated it releases glutamate on neurons in the nucleus of the solitary tract which then releases glutamate onto neurons in the locus coeruleus. When the locus coeruleus is activated in this way, it releases norepinephrine that binds to adrenergic receptors in the basolateral amygdala. Disrupting any component of this functional circuit prevents arousal from enhancing memories formed in other learning and memory systems (McIntyre et al., 2012). Importantly, this neurocircuit would not be necessary for dopamine-mediated memory modulation that is likely to also occur during emotional events.

Exposure to a substance like amphetamine will produce a large release of dopamine in a variety of brain areas (mesolimbic and nigrostriatal for example) and this is probably the main driver of the modulation of memories simultaneously acquired in these regions. This is consistent with the idea that stress hormones and/or amygdala are not required for memory modulation.

Demonstrations that direct infusions directly into the hippocampus modulates hippocampal-dependent memories (for review see Packard and Cahill, 2001) are also consistent with this idea. As noted above, in a related experiment from our laboratory, we have shown that rats with large amygdala lesions show a normal post-training memory improvement effect on a hippocampal-based task when amphetamine is administered peripherally. As the memory improvement in this experiment was not attenuated at all we must assume that there is another mechanism by which hippocampus memories can be modulated when dopamine release occurs independent of the amygdala. Another puzzle for us is our demonstration that rats with large amygdala lesions show normal acquisition of the spatial version of the water task and normal long-term retention and extinction rates. Further, rats with large amygdala lesions showed normal acquisition of a version of the Morris water task that requires rapidly acquired spatial positions (new spatial position each day) and they showed normal rapid spatial learning and long-term retention of this task which is thought to place a high demand on hippocampal processing. Presumably, these water tasks elicit stress and stress hormone release so amygdala modulation mechanisms should be activated and impacting the strength of the memories but this does not seem to be the case (McDonald et al., 2007a).

It is also important to note that stressful experiences in addition to causing stress hormone release also trigger dopamine release in cell groups that project to various learning and memory systems including the hippocampus suggesting to us the possibility that this neurotransmitter release alone might be sufficient to modulate memories in learning and memory systems independent of the amygdala’s role in these functions.

Finally, one final issue surrounding the role of the amygdala in memory modulation is the idea that, in the intact brain, the amygdala may still have a role in the memory modulatory effects of amphetamine on hippocampal plasticity. Consistent with this idea is demonstrations that direct administration of amphetamine in the amygdala alters hippocampal memory but how can this finding be reconciled with the present view and findings. Is the modulatory function of the amygdala sufficient but not necessary for the effects of amphetamine on the hippocampus or is something else going on here?

Our view, through the lens of the encoding and storage view of amygdala function is that post-training amphetamine injections directly into the amygdala during acquisition of a hippocampal-dependent task should probably not influence these memories and would more likely impact amygdala-dependent memories like stimulus-reward learning. Alternatively, even though the spatial version of the Morris water task, for example, is thought to be a hippocampal-dependent task it is possible that early in training there is an amygdala component that although not necessary for normal acquisition or expression of the memory it can influence behavior. Manipulations of the amygdala, like intra-amygdala amphetamine injections might modulate that representation which might improve performance, but it might not be because of a direct influence on the hippocampus. We reported a related finding in which we found that post-training amphetamine injections into the amygdala during training of a task dependent on the dorsal-lateral striatum did not improve performance on that task but did enhance an incidental memory acquired during that task mediated by the amygdala (Holahan et al., 2005).

Taken together, we see this pattern of results as support for the idea that there are multiple memory modulation pathways but clearly, further research is required in this area.

### 4.3. Caveats

One limitation of the current experiment is that dendritic spines were not evaluated. This issue could not be mitigated and is a potential weakness of the conclusions of the present study. It is possible that, in the absence of amygdala function, although amphetamine-induced dendritic branching and length were still altered, spines may not have changed in the nucleus accumbens and prefrontal cortex.

Although possible, it is important to note that correlations between the dendritic changes we examined and dendritic spine changes are very high. We have looked at a wide range of drugs, including stimulants and opiates, and in every case the spines and dendritic changes were highly correlated [see review by Robinson and Kolb (2004)]. However, further research is required to assess spine changes using the procedures reported in the current study.

A second caveat of the present work is the sample size is relatively small. This fact in normal circumstances particularly when a new effect is being reported is of potential concern. However, in the present case previous work using very similar amphetamine exposures and anatomical procedures produce almost identical results to the ones reported in the present report and these kinds of effects have been replicated repeatedly for the last 30 years. Second, the effects were identical in the amphetamine exposure groups which is unusual if these were spurious effects as the two groups combined would be more than sufficient to produce a reliable effect for these kinds of experiments. Finally, many of the classic paper (for example: Robinson and Kolb, 1997) in this area used small group numbers. In that paper, for example, there were 5 subjects per group and other papers from this group have used larger and smaller sample sizes with similar effects. This suggests to us that the effects are quite powerful and reliable.

Another caveat of the present work is the technical malfunction with the behavioral monitoring equipment that resulted in significant amounts of locomotor data being lost. This was unfortunate as it would have been fascinating to see if there was any difference in locomotor activity response to the repeated amphetamine injections amongst the groups. However, this was not the case on day 2 and even if there were differences in locomotor activity in the different groups during amphetamine administration it did not result in a change in the dendritic alterations in the brain regions we assessed. However, since this group was not run in the present experiment more research is required to see if this is the case.

Another potential caveat of the interpretation of the results presented in this report is that amphetamine increases locomotor activity, and this alone could account for the changes in morphology. To address this issue, in one of our previous studies (Robinson and Kolb, 1999) we housed rats in home cages with running wheels as a control for the locomotor activity generated in the drug treatment groups to see if movement produced the changes in dendritic morphology. They had access to the wheels 24 h a day for 4 weeks and injected with saline each day and ran on average 5.43 km per day. For comparison, a rough calculation of the distance traveled (cage crossovers) during an average test session with the stimulant drugs the experimental subjects moved 0.14 km/1.5 h test session. Importantly, there was no effect on dendrites in the regions we assessed in the present study suggesting that it is not the locomotor activity that is inducing the dendritic changes following amphetamine exposures.

Finally, the experiments do not account for sex as a biological variable. This is a weakness, and our future experiments will include this variable.

## 5. Conclusion

The present report presents several important findings concerning the role of the amygdala in modulation of plasticity processes. These demonstrations show that an intact amygdala is not necessary for dendritic plastic changes found in the nucleus accumbens and prefrontal cortex following chronic amphetamine exposure. These results support the idea that modulation of plasticity processes in the brain utilizes multiple pathways.

## Data availability statement

The raw data supporting the conclusions of this article will be made available by the authors, without undue reservation.

## Ethics statement

This animal study was reviewed and approved by the Animal Care Committee, University of Lethbridge.

## Author contributions

RM and BK conceived the experiments and supervised the completion of the work. NH and CG ran the experiments and completed the histological analysis. BK drew the neurons. RM wrote the manuscript with BK editing. All authors contributed to the article and approved the submitted version.
